# The geography of agricultural input markets in rural Tanzania

**DOI:** 10.1007/s12571-021-01181-9

**Published:** 2021-06-26

**Authors:** Pieter Rutsaert, Jordan Chamberlin, Kevin Ong’are Oluoch, Victor Ochieng Kitoto, Jason Donovan

**Affiliations:** 1 International Maize and Wheat Improvement Centre (CIMMYT), P.O. Box 1041, Nairobi, 00621 Kenya; 2 International Maize and Wheat Improvement Centre (CIMMYT), Texcoco, Mexico

**Keywords:** Agro-dealer, Market access, Spatial analysis, Rural development, Remoteness

## Abstract

The expansion of agro-dealers into remote areas can be seen as conducive to more smallholders adopting new technologies and inputs, to include improved seed and fertilizer. However, lower travel costs may be offset by agro-dealer decisions on stocking and pricing, reflecting both travel time from wholesale markets as well as the level of competition in localized areas. This paper investigates the geographical distribution of agro-dealers and related patterns of local market competition on the availability and prices of maize seed and fertilizer. We use a unique census of agro-dealers in eight districts of Tanzania (*n* = 299) which maps distribution points for agricultural inputs in these areas. Results suggested that despite a high number of agro-dealers, almost 30% of farmers lived more than an hour travel time from at least one agro-dealer. Instead of wide geographical coverage, agro-dealers tended to be found in clusters, with strong variation in cluster sizes between different districts. Overall, more remote agro-dealers faced less competition, resulting in fewer stocked product choices and charging higher prices to customers, even after controlling for travel time from district headquarters. Remote farmers are disadvantaged in their uptake of new technologies and critical production inputs due to lack of competition among agro-dealers. Our results suggest that highly aggregated and/or simplified measures of market access fail to reflect important heterogeneity in the market access conditions faced by farmers; a better understanding of distribution networks and competition is needed.

## Introduction

The increased use of inorganic fertilizer, improved seed and other agricultural inputs are critical components of strategies aimed at raising farm productivity and living standards in sub-Saharan Africa (SSA). Authors have attributed the low adoption of improved technologies in African agriculture to various factors, including market imperfections and failures in supply systems (e.g. Duflo et al., 2011; Minten et al., 2013) and low farmer demand, due to low expected benefits from the uptake of such inputs (e.g. Burke et al., 2017; Chamberlin et al., 2020; Duflo et al., 2008; Liverpool-Tasie et al., 2017; Marenya & Barrett, 2009; Michler et al., 2019; Suri, 2011). The dominant model of input supply as pursued by national governments and development partners is one where small-scale, locally based input distributors, commonly known as agro-dealers, provide the point of access for smallholders (Makinde & Muhhuku, 2017; Odame & Muange, 2011). Maize seed companies, for example, depend heavily in agro-dealers for seed distribution, thus avoiding the high costs involved in selling directly to a sparsely populated farming population, connected by a weak road infrastructure, who purchase small quantities of seed only once or twice a year (Langyintuo et al., 2010; Rutsaert & Donovan, 2020). Therefore agro-dealers play a pivotal – if understudied – role to provide farmers with (i) affordable and convenient access to yield-enhancing technologies, and (ii) technical advice on how to use these technologies for maximal economic returns (Allgood, 2011).

Despite the importance of agro-dealers in the input supply system, limited attention has been given to systematically describing farmer access to inputs and distribution of input providers. While the economic remoteness of much of the region is well documented (e.g. Chamberlin & Jayne, 2013; Christiaensen et al., 2003; Headey et al., 2018; Stifel et al., 2016; Stifel & Minten, 2008), these stylized facts provide little direct insight into the spatial distribution of market access across the farming population, and how market accessibility, in turn, correlates with local measures of the structure, conduct and performance in rural input supply markets. In their study on fertilizer usage by smallholders in Burkina Faso, Koussoubé and Nauges (2016) found that fertilizer usage was generally profitable at prevailing prices, even at low investment levels, and concluded that supply constraints via local inputs markets were likely inhibiting adoption. Zavale et al. (2020) found that the growth of the fertilizer industry in Mozambique was limited by demand side factors (i.e., limited effective demand from farmers) and supply side factors (including high transactions costs). Both of these factors are known to have strong spatial expressions (Minten et al., 2013).

How agro-dealers are organized across space – with respect to farmers as well as with respect to one another – is important for a number of reasons. First, such disaggregated spatial patterns are more informative about actual access conditions faced by farmers than aggregate statistics (e.g. ratio of agro-dealers to farmers in a country). Second, the local configuration of agro-dealers tells us something about local input supply choices available to neighboring farmers. Third, the local configuration of agro-dealers may inspire local competition (or lack thereof) which has potential implications for agro-dealer pricing and stocking decisions, which in turn affect farmer input acquisition options. This paper responds to this gap, with a spatially explicit assessment of how smallholder access to maize seed and fertilizer is shaped by the spatial distribution and characteristics of agro-dealers in rural Tanzania.

### Spatial distribution of agro-dealers

As noted, there is an existing empirical literature documenting relatively poor levels of infrastructure and correspondingly long distances that African farmers must travel to access input as well as output markets (Chamberlin & Jayne, 2013; Christiaensen et al., 2003; Stifel et al., 2016; Stifel & Minten, 2008). More remote areas are associated with lower rates of market participation, lower rates of technology adoption, higher input prices and lower output prices, and greater market price volatility. Evidence suggests that farmers’ input usage is strongly conditioned by their access to input markets. Haggblade et al. (2017) found that supply-side changes helped to explain the growth of Mali’s herbicide market, particularly growth in the number of sellers, number of marketed herbicide products, and lowering prices. Tamru et al. (2017) reached similar conclusions for Ethiopia. Farrow et al. (2011) used spatial analysis to determine optimal locations for new agro-dealers in Malawi to improve farmer access to inputs in underserved areas. To shorten the distance between farmers and input suppliers, the Alliance for a Green Revolution in Africa (AGRA), has supported the rural expansion of agro-dealer networks across SSA (Bigirwa & Kapran, 2017). However, Nagarajan (2015) as well as Odame and Muange (2011) found that higher concentrations of agro-dealers in Mozambique and Kenya were found in areas with more intensive production of high value commercial crops and higher input demand. This relates to first hypothesis tested in this study:

- *Hypothesis 1: There is pronounced spatial heterogeneity in the distribution of agro-dealers*

Relatively few studies have evaluated how the characteristics of input markets vary across space. A study by Minten et al. (2013) in Ethiopia indicated that the nature of input supply markets changed strongly with remoteness: prices increase and selection declines. Benson and Mogues (2018) reported strong price increases for fertilizer in Mozambique, Tanzania, and Uganda, which responded, in part, to high internal road transportation costs within the region (Ncube et al., 2016). Yet more explicit attention to how the distribution of agro-dealers is associated with spatial patterns in market access and market conditions has not been examined. One of these factors is the level of competition or market concentration of agro-dealers and choice farmers have between input providers. To the best of our knowledge, the effect of concentration of input suppliers has not been researched in developing countries. Chamberlin and Jayne (2013) assume that competition and trader concentrations are important dimensions in rural markets, although also acknowledge that information on this is rarely collected in smallholder surveys. One can assume that this also holds true for input markets. Collecting complete census of data on the locations and characteristics of agro-dealers in eight districts of Tanzania allows to take the effects of competition into account and test the following hypotheses:

- *Hypothesis 2: The number of agro-dealers serving a rural area decreases with travel time to the nearest urban center (*i.e. *there is decreasing local competition)*
- *Hypothesis 3: Input prices increase with decreasing competition, after controlling for travel time to the nearest urban center*
- *Hypothesis 4: The range of input choices decreases with decreasing competition, after controlling for travel time to the nearest urban center*

With some caveats, the results from this study generally support each of these hypotheses. Our analysis implies that aggregate statistics, such as number of agro-dealers per rural household, or average travel time to the nearest agro-dealer, obscure important variation in access distribution. Furthermore, our results underscore the fact that input market characteristics can vary substantially across gradients of concentration and competition.

The rest of this paper is organized as follows. Section 2 contextualizes our study with a descriptive overview of agricultural input markets in Tanzania. We describe our analytical approach, including our data and methods, in section 3. Our results are presented in the next section in relation to the four hypotheses (section 4), followed by the concluding remarks (section 5).

## Input sector development in Tanzania

The Tanzania Seed Company was the sole supplier of seed until its collapse in the late 1980s with market liberalization. Following market liberalization, multinational and regional seed companies emerged, and have been actively involved in the Tanzanian seed market. Other seed producers also appeared, including farmer groups and NGOs, but which focused on producing and distributing improved open-pollinated varieties (De Groote et al., 2002). Currently, 40 active maize seed companies operate in the country (Mabaya et al., 2017), and private sector provides 80% of the formal seed supply (Ashley, 2016). However, much of the certified maize seed used is not produced in Tanzania (ASARECA/KIT, 2014). Of the seed supplied by the private sector, 85% is imported by multinational companies with large South African and/or Zambian production sites (Ashley, 2016). Most smallholders access improved varieties, fertilizer and other inputs through direct purchase from private agro-dealers (Lyimo et al., 2014).

A major push towards increased use of seeds and fertilizers in Tanzania was provided by the National Agricultural Input Voucher Scheme (NAIVS), supported by The World Bank. NAIVS was a smart input subsidy program that was piloted in the 2007/08 season in two districts (World Bank, 2014). The program was launched at a large scale from 2008/09 as a response to the sharp rise in global grain and fertilizer prices in 2007 and 2008. Farmers who met the criteria of the program could purchase limited quantities of fertilizer (basal and top dress) and maize or rice seed with a 50% subsidy through input vouchers that could be redeemed at local agro-dealer stores. During these years, NAIVS was a major outlet for seed companies to sell certified seed: seed companies reported selling 46% of their maize seed through the NAIVS program (Mabaya et al., 2017). The World Bank support for the NAIVS program ran until the 2013/14 season but after a year without support, the government brought the program back in 2015/16, although at a smaller scale (Lewis & Masinjila, 2018; Mather & Ndeytabula, 2016).

A recent assessment of the program (2014) showed that subsidies had a significant effect on the rice and maize productivity in Tanzania and through the program 2400 agro-dealers were trained on seed and fertilizer management and business practice. On the other hand, the program also dealt with logistical challenges such as farmers receiving their vouchers too late, delayed payments for seed and fertilizer suppliers as well as the agro-dealers participating in the program. Nonetheless, the NAIVS program succeeded in introducing improved seed and fertilizer across the country, with 74% of the farmers continuing to purchase seed and 19% continuing to purchase fertilizer (World Bank, 2014).

From 2017, the government initiated the Fertilizer Bulk Procurement System (FBPS) under the supervision of the Tanzania Fertilizer Regulatory Authority (TFRA). Instead of a subsidy system, this program focuses on two types of fertilizer, urea and diammonium phosphate (DAP), and organizes bulk sales through competitive bidding and aims to make fertilizer more affordable for farmers. One of the challenges with subsidized fertilizer programs has been the higher-than expected margins taken on wholesale and agro-dealer level, with farmers experiencing little in terms of lower input prices (Cameron et al., 2017). Therefore, the government decided to work with indicative wholesale and retail prices, regulated by the TFRA, and stronger border regulation to reduce smuggling. Cameron et al. (2017) highlighted that this approach would also present new problems due to increased government regulation and interference in the supply chain.

## Material and methods

### Research area and sampling

**Fig. 1:**
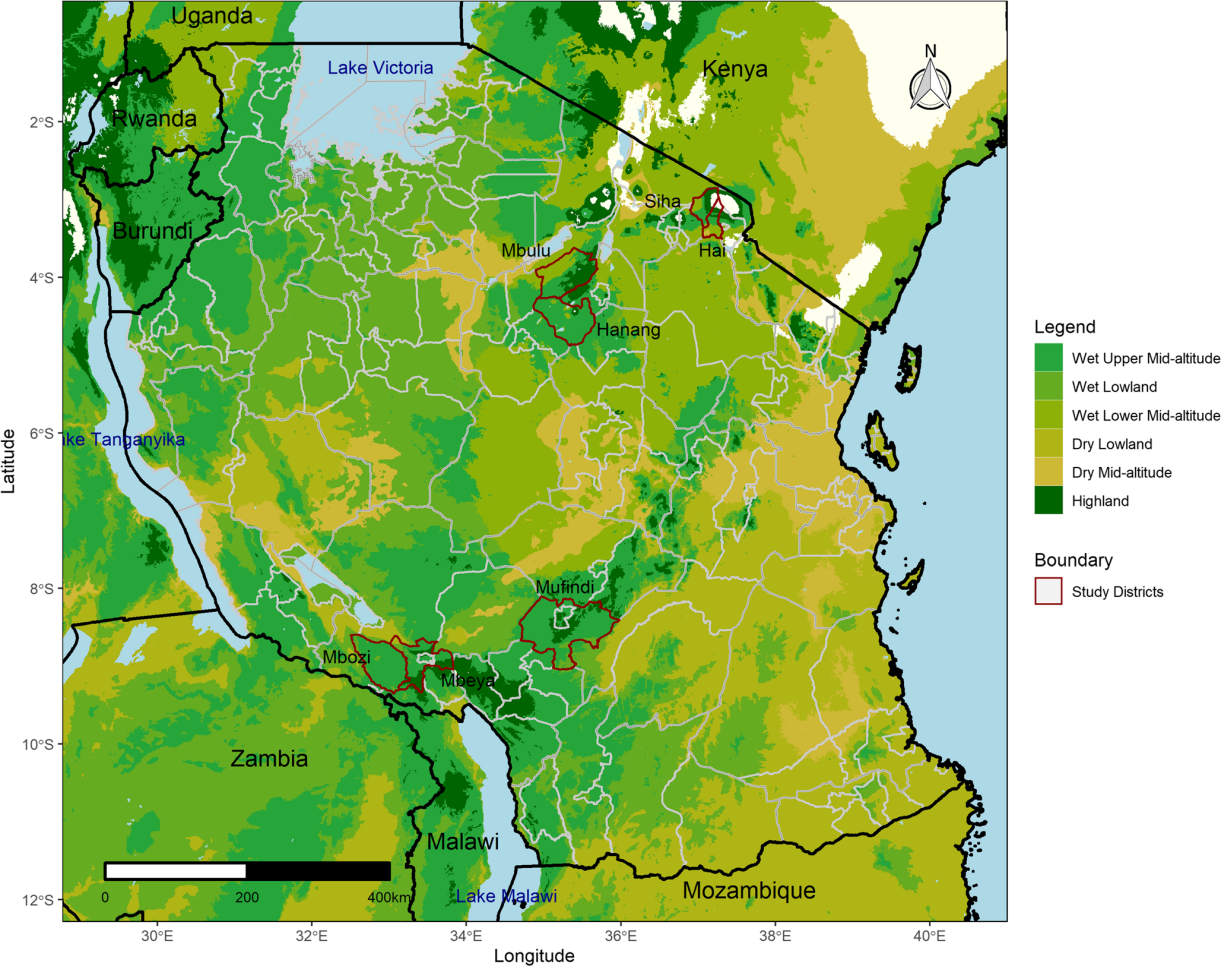
Sampled districts in Tanzania

A survey was conducted with 299 agro-dealers between September–October 2019. A thorough attempt was made to interview all the agro-dealers in operation which were located in the selected districts. The eight districts included were Hai and Siha (Kilimanjaro region), Hanang and Mbulu (Manyara region), Mbeya urban and rural^1^ (Mbeya region), Mufindi (Iringa region) and Mbozi (Songwe region since 2016; previously part of Mbeya region) (Fig. 1). These districts were selected purposively to represent a broad range of market access and agroecological growing conditions within the Tanzanian maize belts of the Southern Highlands and Northern zones.

To ensure total agro-dealer coverage, the sample selection protocol was applied in a similar manner across each district, with support from the Tanzanian Agricultural Research Institute (TARI). On arrival in each district, a meeting was organized with local District Agriculture and Livestock Development Officer who maintained a list of agro-dealers active within the district. Based on the agro-dealer spread per district, two data collection teams of each four enumerators and one team leader, divided the work and further liaised with ward or village level agricultural officers to confirm the original agro-dealer list and provide logistical support (i.e. make appointments with agro-dealers for the interview as well as accompanying the team on the store visits). Per town or market, the data collection team interviewed agro-dealers that were active in 2019. During these interviews, the agro-dealer lists were also checked by the participants to check for any mistakes or missing agro-dealers they knew of. When corrections were necessary, the team would make appointments with agro-dealers that were not on the list and carry out all remaining interviews before travelling to the next district.

### Survey implementation and design

Interviews were carried out with the individual who was most knowledgeable about maize seed sales and day-to-day seed-related transactions. This selection was based on some screening questions. More than half of the respondents were the store owner (55.5%), 39.8% were experienced employees and 4.7% identified themselves as store managers. Interviews lasted roughly 60 min and information was recorded with tablets by experienced enumerators.

The survey covered current stock, pricing and sales of maize seed and fertilizer, among other topics. It was extensively pretested and revised over several iterations. The questions included a mix of closed questions and more interactive questions. Flash cards were used in some cases to facilitate engagement with interviewees for multiple choice questions.

### Analysis of agro-dealer clusters and travel time

Analysis was carried out on two levels: agro-dealer level and agro-dealer cluster level. Agro-dealer clusters considered all agro-dealers that were located within a 1 km radius. Clustering was done through a two-step process. First, the statistical package in R was used to automatically cluster all agro-dealers within several predefined radii: 500 m, 1 km, 2 km and 5 km. The software initially considers all the agro-dealers as clusters. Then it incrementally joins the two closest agro-dealers into one cluster, until all agro-dealers close enough to be in a circle of the predefined radius are clustered together. An evaluation of the predefined radii showed that 500 m was too narrow, with clusters being positioned right next to each other, however 2 km was already quite broad, sometimes covering two markets or towns. Therefore, 1 km radius was selected as the preferred cluster-size. Secondly, the team went over each cluster to see if there weren’t any clusters that needed to be separated or merged. Three manual corrections (all mergers) were carried out to finetune the clusters. Table 1 reports the different cluster sizes and number of clusters in the study sample. Cluster sizes ranged from 1 up to 34 agro-dealers in a single cluster. 18.1% of the agro-dealers had no competition and 22.4% had one or two competitors. There were 4 agro-dealer clusters with over 10 competitors, which accounted for 26.4% of the total agro-dealer sample.

**Table 1:** Agro-dealer clusters (based on 1 km radius) in 8 districts in Tanzania

Cluster size	Number of clusters	Total # of agro-dealers per cluster size	
1	54	54	No competition (18.1% of agro-dealers)
2	20	40	1–2 competitors (22.4% of agro-dealers)
3	9	27	
4	2	8	3–5 competitors (15.4% of agro-dealers)
5	4	20	
6	3	18	
7	1	7	6–10 competitors (17.7% of agro-dealers)
8	2	16	
9	1	9	
10	1	10	
11	1	11	
12	1	12	> 10 competitors (26.4% of agro-dealers)
14	1	14	
19	1	19	
34	1	34	
Total	102	299	

Note: The number of competitors is one less than the cluster size. For example, in a cluster of 3, each agro-dealer has 2 competitors within that cluster. Source: Agro-dealer survey data collected by authors in 2019

To approximate the effective distances between farm locations and the nearest agro-dealer, and from agro-dealers to the district headquarters, we employ a geospatial travel time model which uses data on the road network, elevation and landcover to estimate the time it takes to travel from any location within the study area to the nearest agro-dealer. This model is similar to models used in other studies (e.g. Chamberlin et al., 2014; Headey et al., 2018; Weiss et al., 2018), and calibrated such that known travel times between locations in the study site are accurately approximated. Data on the road network come from the OpenStreetMap database (OSM, 2021). The model uses a least-cost algorithm, measured in minutes, such that the most efficient route between locations is the basis of the travel time estimate. In this model, farmers are assumed to walk in areas without roads, and to use motorized transportation where there are roads. As such, it may be considered a lower bound estimate of travel times, as it does not account for waiting times or delays in transportation services.

## Results and discussion

### Spatial heterogeneity in input access

Table 2 provides an overview of access to agricultural inputs per district, including: the ratio of agro-dealers to farmers, average farmer travel time to the nearest agro-dealer, number of agro-dealer clusters, distribution of cluster sizes, and shares of agro-dealers stocking different inputs. On average, there was one agro-dealer per 1619 farming households with the highest density in Siha (1:978) and Mbeya (1:1073) and the lowest density in Mufindi (1:2919).^2^ Compared with the average of one agro-dealer per 2900 households in Tanzania reported by Mabaya et al. (2017), the selected regions indicate a more extensive presence of agro-dealers.

**Table 2:** Agro-dealer coverage and input access per district

	Total	Hai	Hanang	Mbulu	Siha	Mbeya	Mufindi	Mbozi
Number of agrodealers	299	23	24	27	27	89	27	82
Agro-dealer / farmer household ratio	1 / 1619	1 / 1870	1 / 2014	1 / 1898	1 / 978	1 / 1073	1 / 2919	1 / 1716
Average farmer travel time to an agro-dealer (minutes)	59	39	57	80	39	42	97	38
% of farmers within 30 min travel time	40%	53%	36%	37%	53%	48%	40%	63%
% of farmers within 1 h travel time	74%	72%	80%	75%	77%	74%	74%	71%
Agro-dealer clusters	102	9	13	8	7	25	18	22
Average cluster size	2.9	2.6	1.8	3.4	3.9	3.6	1.5	3.7
Agro-dealer competition								
No competition (%)	18.1%	21.7%	33.3%	11.1%	3.7%	3.7%	48.1%	13.4%
1–2 competitors (%)	22.4%	17.4%	37.5%	25.9%	25.9%	25.9%	33.3%	20.7%
3–5 competitors (%)	15.4%	21.7%	0.0%	22.2%	40.7%	40.7%	18.5%	9.8%
6–10 competitors (%)	17.7%	39.1%	29.2%	40.7%	29.6%	29.6%	0.0%	0.0%
>10 competitors (%)	26.4%	0.0%	0.0%	0.0%	0.0%	37.1%	0.0%	56.1%
Input availability at agro-dealer (%)								
Maize seeds	100%	100%	100%	100%	100%	100%	100%	100%
Other seeds	68%	87%	83%	89%	89%	62%	70%	50%
Fertilizer	74%	96%	33%	56%	96%	70%	96%	77%
Herbicide	75%	91%	79%	56%	100%	74%	67%	72%
Pesticide	81%	91%	96%	96%	100%	72%	85%	70%
Foliar feeds	65%	83%	79%	74%	85%	58%	67%	51%
Farm tools	52%	52%	54%	59%	78%	47%	59%	44%
Veterinary chemicals	42%	43%	83%	85%	59%	30%	30%	28%
Livestock feeds	19%	35%	29%	22%	11%	18%	26%	11%
# maize seed varieties per store	7.4 (4.6)	9.0 (5.6)	8.4 (3.9)	12.1 (4.7)	9.4 (4.4)	5.7 (4.5)	4.9 (3.2)	7.2 (3.5)
# fertilizer types per store	4.8 (2.2)	3.9 (1.6)	2.9 (1.4)	4.1 (2.3)	4.6 (2.1)	5.1 (2.3)	5.6 (2.9)	5.0 (2.0)

Source: Agro-dealer survey data collected by authors in 2019. Agro-dealer / farmer household ratio is calculated using estimated farm households per district from the 2007/8 Tanzanian National Agricultural Sample Census data, after incorporating estimated rural population growth rates to generate estimated numbers of farm households in 2019

**Fig. 2:**
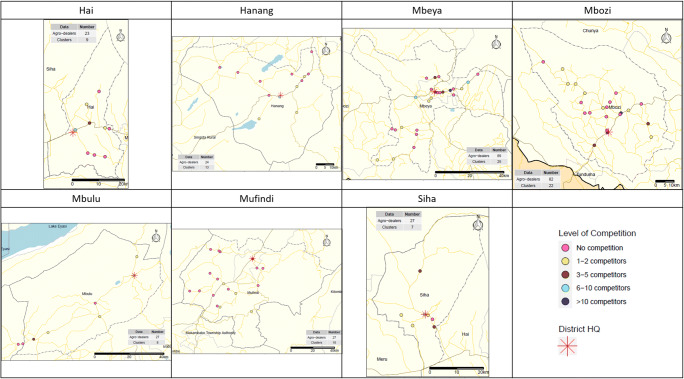
Agro-dealer distribution and level of competition per district

Spatial distribution of the agro-dealer clusters per district are visualized in Fig. 2. Looking closer at the average travel time for farmers to an agro-dealer as well as the percentage of farmers who lived within 30 and 60 min, there was less variation between districts, with the exception of Mufindi and Mbulu. Average travel time across districts was approximately one hour to the closest agro-dealer. For Hai, Siha, Mbeya and Mbozi, the average travel time was approximately 40 min. These places also had the highest shares of the district’s farmers living within 30 min of an agro-dealer. Although the number of agro-dealers per rural household was relatively high, there is pronounced spatial heterogeneity in agro-dealer distribution, confirming *hypothesis one.* As discussed by Odame and Muange (2011), demand factors could be a key in influencing geographical coverage with stronger agro-dealer presence in districts with higher tendency of farmers to invest in agricultural inputs. Overall, three quarters of the farmers in these districts lived within one hour of an agro-dealer. Reducing distances traveled by farmers to obtain agricultural inputs has been one of the main drivers behind development programs for better access to inputs (Adesina et al., 2014; Makinde & Muhhuku, 2017). While a large size of the farmer population live within the proximity of an agro-dealer, these numbers are still low compared to a country like Kenya (Chamberlin & Jayne, 2013).

The average agro-dealer cluster size across all districts was 2.9, but the levels of agro-dealer concentration (or competition) were very diverse between the districts. Agro-dealers in Hanang and Mufindi had the lowest of competition with most of them standing alone or having 1–2 competitors. The opposite was true for Mbulu and Siha, Mbeya and Mbozi. In these districts, most agro-dealers had at least one competitor and in the latter two over 50% of the store had five competitors or more, going up to a cluster of 34 in Mbeya. In Mbozi, more than 50% of all agro-dealers in the district were grouped in two agro-dealer clusters.

Maize seed was available at every agro-dealer, and almost 70% of the stores also sold seed for other crops during the year, but this was more common in the Northern districts (Hai, Hanang, Mbulu and Siha). Agro-dealers offered a range of maize seed varieties, although the choice in store was lower in the Southern districts compared to the Northern districts. This might be due to a higher diversity of agro-ecologies in Northern Tanzania compared to the Southern zone where most selected districts were part of the Southern highlands. In the Southern Highlands, less diversity in varieties is required due to more uniform elevation (i.e. mainly highlands) and rainfall patterns, leading to a strong dominance of late maturity varieties. In the Northern zones, a higher diversity in elevation and rainfall patterns leads to farmer demand for early, intermediate as well as late maturity varieties. Fertilizer availability was rather low in Hanang and Mbulu; on the other hand, veterinary chemicals were widely sold in those two districts, which is in line with Manyara being a pastoral farming region (Chengula et al., 2013). Herbicides as well as pesticides were also widely available across the districts. Agro-dealers had on average five different fertilizer brands or types available in their store.

### Relation between spatial distribution and competition

**Fig. 3:**
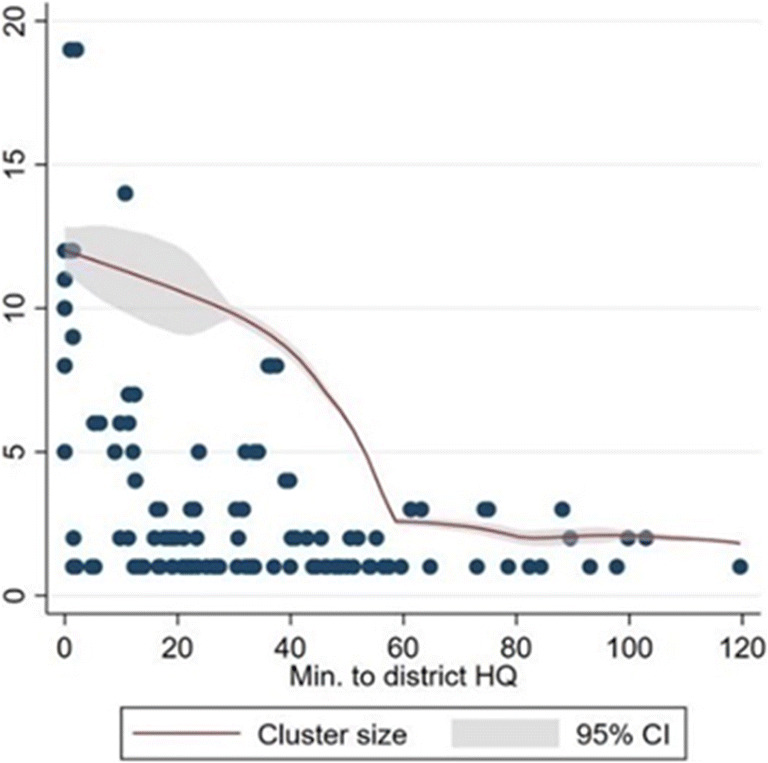
Cluster size by travel time from district headquarters. Source: Agro-dealer survey data collected by authors in 2019

Here we explore the effects of spatial agro-dealer distribution on physical access to inputs. The relation between agro-dealer cluster size and travel time from the district headquarter (HQ) is plotted in Fig. 3. Cluster sizes became smaller further away from the district HQ, supporting *hypothesis two* that remote farmers had less choice between agro-dealers to purchase their inputs unless they invested additional time and resources in travel.

**Fig. 4:**
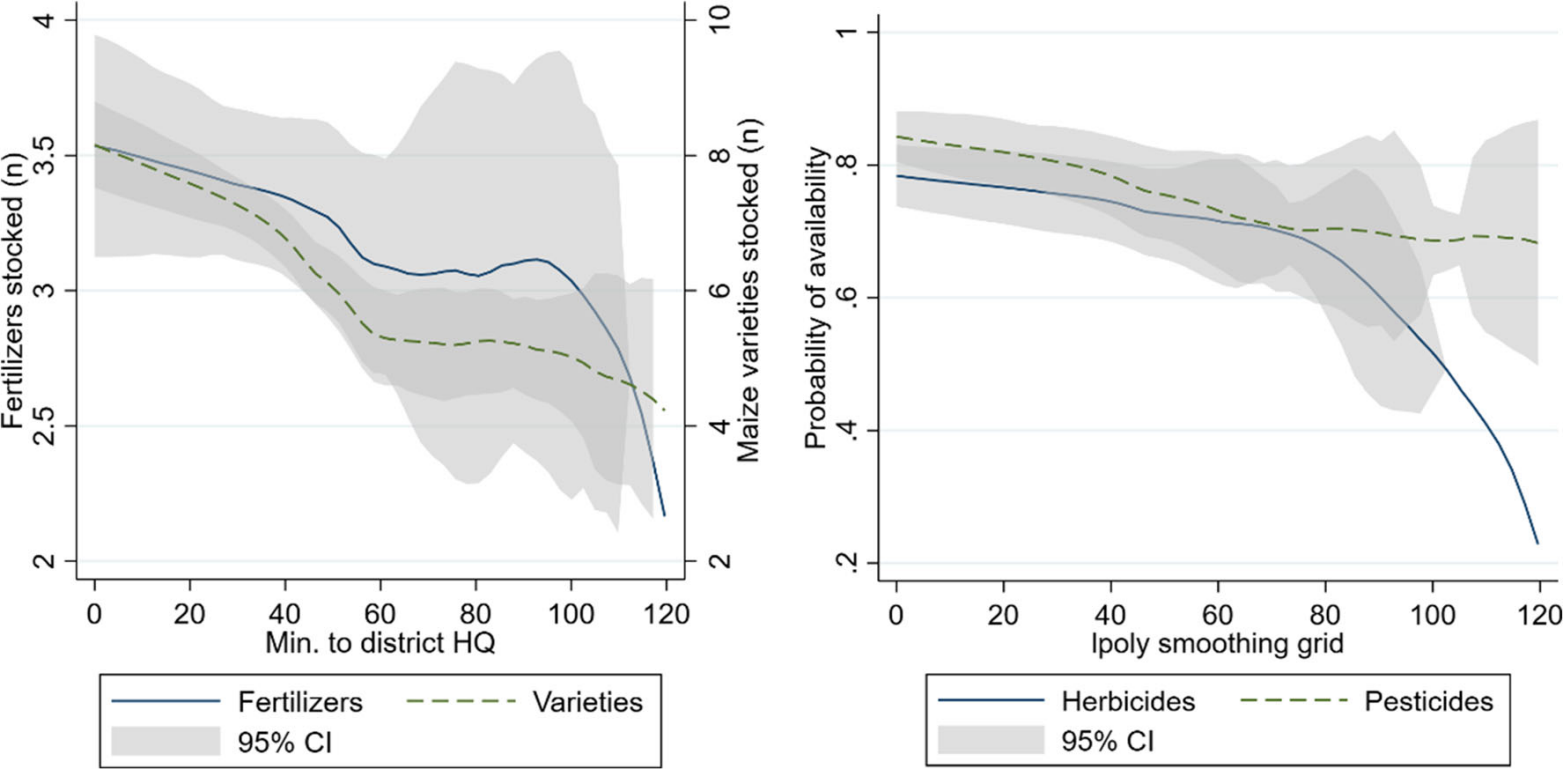
Stock availability indicators, by travel time from district headquarters

Besides choice between agro-dealers, remote farmers also faced reduced input choices. Figure 4 shows that both maize seed as well as fertilizer choices diminished the further away from the district HQ. While stores had on average eight maize seed varieties in stock close to city centers, this dropped below six an hour away from district HQ and declined further with increased travel time. Availability of pesticides and herbicides followed a similar pattern. While chances of finding those products close to the district HQ is high, this likelihood drops further from district HQ, especially for herbicides. These results support the second hypothesis that more remote farmers have fewer choices.

**Fig. 5:**
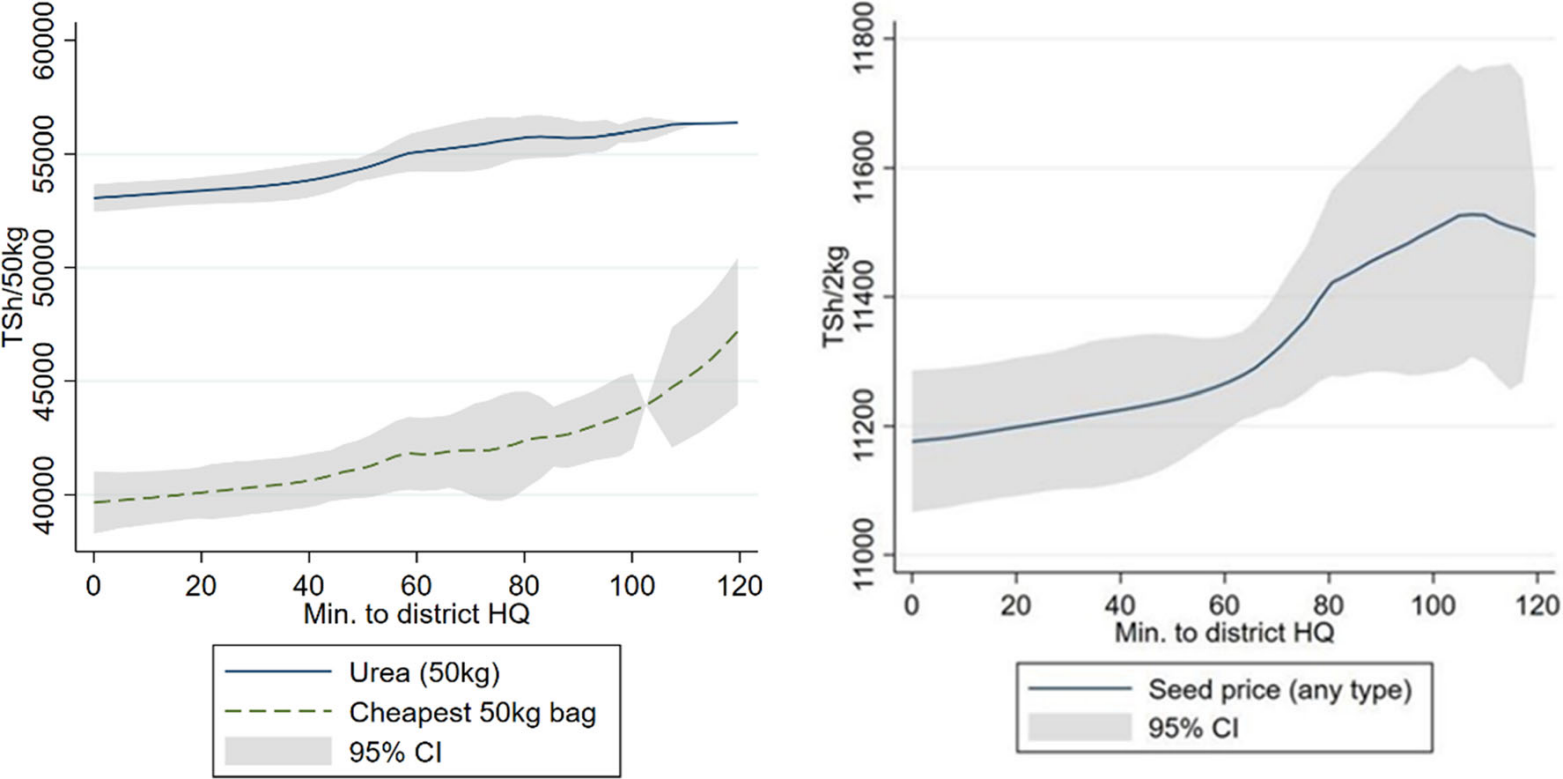
Fertilizer & seed prices by travel time from district headquarters

The opposite results were obtained for the sales price of inputs, more remote farmers paid higher prices for the same inputs. Figure 5 reports the change in average price for urea as well as the cheapest fertilizer option on the left. Both prices were positively correlated with travel time from district HQ. On the left side seed prices are reported.^3^

### The effect of competition on input prices

We specify a number of regression model specifications to evaluate the potential role of agro-dealer competition in pricing. Table 3 shows the results of models in which the dependent variable is the price of a 50 kg bag of fertilizer. We show results for the four most commonly used fertilizer types – urea, diammonium phosphate (DAP), ammonium sulfate (SA) and calcium ammonium nitrate (CAN) – as well as the least expensive fertilizer in stock at any given agro-dealer. (Because the latter mixes different types of fertilizer, it should be interpreted with care, as we are not holding type constant.) For each of these, three specifications are shown: 1) controlling only for travel time from the district headquarters (which is typically the primary wholesale entrepot through which inputs pass before reaching the agro-dealer location^4^) and for cluster size; 2) additionally controlling for other agro-dealer characteristics^5^ and district fixed-effects; and 3) including an additional interaction term that takes a value of one for areas further than 30 min from the nearest district headquarters. The latter specification is designed to measure whether or the correlation of prices with cluster size differs between relatively accessible versus more remote areas.^6^

**Table 3:** Correlates of retail fertilizer price (TSh/50 kg bag)

	(1)	(2)	(3)	(4)	(5)	(6)	(7)	(8)	(9)	(10)	(11)	(12)	(13)	(14)	(15)
	Urea	Urea	Urea	DAP	DAP	DAP	CAN	CAN	CAN	SA	SA	SA	Least expensive	Least expensive	Least expensive
Min. to district	21.73**	12.79	22.33*	4.867	5.173	19.58	22.26	7.474	18.09	8.789	5.685	21.25	27.19	9.732	46.38
HQ	(9.294)	(12.25)	(13.29)	(11.44)	(13.36)	(13.69)	(14.20)	(15.99)	(17.20)	(23.99)	(25.20)	(28.19)	(25.19)	(26.40)	(28.35)
Cluster size	−100.6***	−82.56***	−80.67***	−75.48***	−19.61	−16.84	−23.74	−7.107	−4.892	−71.70***	−50.73**	−45.64*	−112.6***	−16.31	−12.66
	(14.99)	(17.74)	(17.49)	(18.77)	(31.89)	(31.62)	(22.21)	(31.95)	(32.12)	(21.31)	(24.31)	(24.71)	(39.91)	(48.59)	(51.04)
Cluster size * [remote==1]			−216.2			−330.2**			−270.3**			−257.6			−821.8***
			(140.0)			(133.8)			(115.9)			(170.9)			(247.2)
Firm controls	No	Yes	Yes	No	Yes	Yes	No	Yes	Yes	No	Yes	Yes	No	Yes	Yes
District FE	No	Yes	Yes	No	Yes	Yes	No	Yes	Yes	No	Yes	Yes	No	Yes	Yes
N	193	193	193	191	191	191	160	160	160	144	144	144	220	220	220
R^2^	0.160	0.279	0.285	0.073	0.203	0.213	0.084	0.233	0.242	0.130	0.272	0.277	0.074	0.210	0.229

Source: Agro-dealer survey data collected by authors in 2019. Notes: Models estimated on the sample of agro-dealers which sell fertilizer. [remote==1] is a dummy indicator areas more than 30 min by motorized transport from the district headquarters. Firm-level controls include years in business, an indicator of whether other shops are operated by the same owner, and number of employees. An indicator is also included for shops which indicated that urea and DAP price decrees affected their pricing decisions. Dependent variable is the retail price of a 50 kg bag of fertilizer, in Tanzanian shillings. DAP = diammonium phosphate; SA = ammonium sulfate; CAN = calcium ammonium nitrate. “Least expensive” denotes the price of the least expensive 50 kg bag of fertilizer stocked by an agrodealer, regardless of type. Heteroskedasticity robust standard errors shown in parentheses; significance levels denoted as 0.01 *** 0.05 ** and 0.1 *

Unsurprisingly, the selling price increases with travel time in all cases and decreases with cluster size, although these coefficient estimates differ in magnitude and significance across specifications. After controlling for other firm characteristics and district-level fixed effects (which account for 30% of the variation in cluster size), we found that the magnitude of the coefficient estimates for cluster size decreases, but remains highly significant for the urea and SA price models. These results are consistent with lower prices being affected by local agro-dealer competition, as proxied by cluster size, even after controlling for travel time and other geographic controls.

When we additionally interact the cluster size with a dummy indicator for relatively remote area (in specifications 3, 6, 9, 12 and 15), we see that the magnitude of the coefficient estimate for cluster size is much larger (and negative) in more remote areas in all specifications (although only highly significantly so in the DAP, CAN, and least expensive fertilizer models). This result suggests that competition effects are likely to be more important in relatively isolated settings. (We also tested for non-linearities in cluster size, but did not find strong evidence for any such effects and therefore do not report the results of those specifications here.)

As discussed in section two, above, beginning in 2018, Tanzania introduced a system of “indicative” local retail prices for urea and DAP, defined at local levels – either at district or ward levels – by the TFRA. Only 21% of agro-dealers indicated that these indicative prices had any impact at all on their pricing or stocking decisions. Nonetheless, to control for this, we included an indicator variable for those who said that their pricing decisions were affected. While our results were not sensitive to the inclusion of this control, we maintained it in the specification with district and firm-level controls.

The magnitude of the estimated correlations varies by fertilizer type and specification. Using specification (2) for urea, the most important fertilizer in the region, each additional competitor within a cluster was associated with a 83 TSh drop in price, after controlling for travel time. This was equivalent to just 1.5% of the average price of a 50 kg bag of urea. However, the difference between a one-agro-dealer cluster and a cluster of 12 (which occurs at the 75th percentile of our sample) was 18% -- no longer a trivial difference.

We similarly estimated the conditional correlation of maize seed prices (Table 4). Similar to the fertilizer price results, seed prices increased with travel time and decreased with cluster size. Here also, however, the significance of these results was attenuated by the addition of firm- and district-level controls. Note that we pool seed varieties in this outcome and cannot rule out the possibility that agro-dealers preferentially stock cheaper varieties in more remote and less competitive locations. These findings generally supported *hypothesis 3*, i.e., that input prices increase with decreasing competition, after controlling for travel time to the nearest urban center.

**Table 4:** Correlates of maize seed price (TSh/2 kg bag)

	(1)	(2)	(3)
Min. to district	1.173	2.643	3.922
HQ	(2.624)	(2.528)	(2.775)
Cluster size	−19.61***	−3.941	−3.920
	(3.987)	(4.822)	(4.978)
Cluster size			−34.55
* [remote==1]			(25.27)
Firm controls	No	Yes	Yes
District FE	No	Yes	Yes
N	299	299	299
R^2^	0.058	0.253	0.256

Source: Agro-dealer survey data collected by authors in 2019. Notes: [remote==1] is a dummy indicator areas more than 30 min by motorized transport from the district headquarters. Firm-level controls include an indicator of association membership, and number of employees. Heteroskedasticity robust standard errors shown in parentheses; significance levels denoted as 0.01 *** 0.05 ** and 0.1 *

### Agro-dealer competition and input choice

We applied this framework as well to examine the correlation between local competition and input choices available to farmers. Table 5 presents regression estimates for (1) the number of fertilizers available; (2) the number of maize seed varieties available; (3) the availability of herbicides, and (4) the availability of pesticides. The latter two are binary outcomes and the models can be interpreted as linear probability models. In these specifications, we controlled for firm-level characteristics and district fixed effects. (Specifications with remoteness interaction terms did not indicate significant interaction effects and are not shown here.) Across all outcomes, the coefficient estimates for travel time were negative – indicating declining choice with travel time – and positive for cluster size – indicating expanding choice with competition, providing some support for our *4th hypothesis*. (Note that these outcome variables are defined at the agro-dealer level, so this indicates increasing choice at any given outlet, i.e., in addition to the increased choices implied by multiple dealers with differing stocks in a given location.).

**Table 5:** Indicators of stock availability

	(1)	(2)	(3)	(4)
	Number of fertilizers available	Number of varieties available	1 = herbicides available	1 = pesticides available
Min. to district	−0.0129***	−0.0239**	−0.00161	−0.00250**
HQ	(0.00484)	(0.00983)	(0.00140)	(0.00115)
Cluster size	−0.0152	0.0840***	0.00230	0.00160
	(0.0142)	(0.0155)	(0.00247)	(0.00252)
Firm controls	Yes	Yes	Yes	Yes
District FE	Yes	Yes	Yes	Yes
N	299	299	299	299
R^2^	0.233	0.342	0.135	0.167

Source: Agro-dealer survey data collected by authors in 2019. Notes: Firm-level controls include an indicator of association membership, and number of employees. Heteroskedasticity robust standard errors shown in parentheses; significance levels denoted as 0.01 *** 0.05 ** and 0.1 *

## Conclusions

Although the literature on input markets and value chains in developing countries is large, one aspect of input supply markets that has been particularly neglected is how agro-dealers are distributed across geographic space, and how that spatial distribution correlates with other characteristics of market access, such as pricing and stock availability to smallholder farmers. This study has addressed this gap by examining the spatial configuration of rural input markets in Tanzania, using a census of agro-dealers in eight districts, representing a broad range of production and market conditions in the Southern Highlands and Northern zones, which are the major maize producing regions of the country. We found pronounced spatial heterogeneity in the distribution of agro-dealers across geographic space within districts, even after the distribution of rural populations was accounted for. We also found that the number of agro-dealers serving a rural area decreased with travel time to the nearest urban center, an indicator of decreasing local competition as markets attenuate with travel time. This translated into a decreased range of input choices and higher input prices available to farmers in more remote areas. Our finding that these market characteristics were associated with input dealer cluster size, even after controlling for travel time to the nearest urban center, suggested that local market competition effects were in play.

Input producers that rely mainly on agro-dealers for distribution, such as maize seed companies, are most likely influenced by these market dynamics. When dealing with smallholder farmers in remote areas, who are often cash constrained, it is important to consider the affordability of inputs in local outlets. The combined effect of more expensive logistics and lack of competition on seed prices in remote areas might contribute to reduced demand. Moreover, the reduction of seed choices in remote areas has important implications. Agro-dealer networks might not be that useful for bringing new hybrids into remote markets if the varieties which are most popular nationally are those which are most likely to find their way into more remote distribution systems (possibly missing opportunities for better targeting of new varieties to production conditions faced by seed buyers). On the other hand, there might be opportunities in remote markets for local companies who are able to set up shorter distribution chains and promote their seed at a lower cost than the limited offer of input suppliers. A better understanding of seed retail markets and prices would allow stronger territory planning for local seed producers.

Our study has potentially important implications for policy. Our results indicate that efforts to understand input adoption by smallholders should pay greater attention to the geographical structure of input markets, as well as the local market characteristics that vary across this geography. In practice this means acknowledgement of local variations in input market access conditions when defining agronomic recommendations and extension programming (e.g. to avoid making recommendations which are not profitable or even feasible), and to address market supply constraints in integrated agricultural development interventions. Our work complements the empirical literature on input profitability, availability and access to information in sub-Saharan Africa, suggesting that the configuration of agro-dealers plays an important role in such local input market characteristics. More and better information on market characteristics – both from agro-dealer surveys and more granular market information systems – can help to diagnose and address input market constraints where they are most acute. Policy options for improving market access may include spatially targeted efforts to incentivize small-scale business creation, including training, credit or other incentives for agro-dealer investments.

Some limitations of our study are in order. First, our dataset provided a comprehensive measure of agro-dealer distributions for the districts we studied; it was not nationally representative. Expansion of our analysis to additional areas would be valuable and may generate additional insights about the distribution of input markets across the country’s diverse geography. Secondly, we do not consider edge effects – i.e., the possibility that some farmers near the boundaries of districts may be most proximate to agro-dealers in neighboring areas. Given our knowledge of study areas, and the structure of markets relative to district headquarters, we felt that this was unlikely to be of consequence for our analysis. Nonetheless, future studies may address this question more explicitly. Thirdly, we acknowledge that our conclusions about competition effects are only tentative, as we cannot claim to fully control for unobserved factors that may also influence pricing and stocking decisions. Finally, there would be great value in additional empirical analysis which more explicitly links farmer investments and management decisions with the nature of local input supply markets. This would require collection of farm household survey data which is explicitly linked with information about local input market characteristics. Such data do not currently exist, to our knowledge, but could enable detailed evaluations of how farmer decisions are affected by localized market conditions.

## Footnotes

1 In the analysis of the results, Mbeya urban and Mbeya rural are considered as one unit due to their geographical structure with the rural zone almost completely surrounding the urban zone and a large concentration of agro-dealers in the urban zone supplying the rural surroundings.

2 These density calculations were made using district level farming population estimates from 2007/08 National Sample Census of Agriculture (National Bureau of Statistics, 2010). To account for population growth between 2008 and the time of our survey, we use an estimated annual rural population growth rate of 1.8524%, estimated for Tanzania in 2019 in the World Development Indicators database compiled by the World Bank, with national rural population growth estimates based on the United Nations Population Division’s World Urbanization Prospects (2018 Revision).

3 Besides an increase of the average seed price, the standard deviation in prices also showed an upward trend further away from the district HQ, which may indicate increased variability in seed prices. However, our sample size also decreases with travel time from the district HQ, which would contribute to an increased standard deviation, and thus we cannot make definitively conclusions about price variability as a function of remoteness. This would be an interesting avenue for further empirical research.”

4 This has not been captured in quantitative data but comes out of qualitative interviews with agro-dealers as well as government informants.

5 Firm-level controls include years in business, an indicator of whether other shops are operated by the same owner, the number of shop employees, and an indicator of whether the shop manager reported having been affected by TFRA indicative pricing recommendations for urea and DAP. As an anonymous reviewer notes, years in business may serve as a proxy for the quality of input marketing services provided by the agro-dealer.

6 We also include a dummy variable for the community of Haydom, in Mbulu, Manyara Region, which is located near the border with Singida Region, serving farmers on both sides of the border, and qualitatively different in this respect than other locations in our sample, which all primarily serve farmers within the same district. A total of 6 agro-dealers are located in Haydom.

## References

[CR1] Adesina, A. A., Langyintuo, A., Bugo, N., Makinde, K., Bigirwa, G., & Wakiumu, J. (2014). Improving farmers’ access to agricultural inputs and finance: Approaches and lessons from sub-Saharan Africa. In P. B. R. Hazell & A. Rahman (Eds.), *New directions for smallholder agriculture* (pp. 250– 323). Oxford University Press. 10.1093/acprof:oso/9780199689347.003.0009

[CR2] Allgood, J. (2011). *Agrodealer Development in Developing and Emerging Markets.* Paper presented at the AIARD Annual Conference 2011, Washington, DC,

[CR3] ASARECA/KIT. (2014). *Tanzania seed sector assessment: A participatory national seed sector assessment for the development of an integrated seed sector development (ISSD) programme in Tanzania*. ASARECA/KIT.

[CR4] Ashley, E. (2016). *Registering and certifying agricultural inputs in Tanzania: An update assessment of key constraints and recommendations for change*. The Africa Enterprise Challenge Fund (AECF).

[CR5] Benson T, Mogues T (2018). Constraints in the fertilizer supply chain: Evidence for fertilizer policy development from three African countries. Food Security.

[CR6] Bigirwa, G., & Kapran, I. (2017). Setting up seed companies in sub-Saharan Africa. In J. DeVries & Z. Masiga (Eds.), *Seeding an African green revolution: The PASS journey*. AGRA.

[CR7] Burke WJ, Jayne TS, Black JR (2017). Factors explaining the low and variable profitability of fertilizer application to maize in Zambia. Agricultural Economics.

[CR8] Cameron, A., Derlagen, C., & Pauw, K. (2017). Options for reducing fertilizer prices for smallholder farmers in Tanzania. Prepared for the Ministry of Agriculture, livestock and fisheries (MALF), United Republic of Tanzania, June 2016. Policy report. MAFAP (monitoring and analyzing food and agricultural policies). FAO.

[CR9] Chamberlin J, Jayne TS (2013). Unpacking the meaning of 'Market Access': Evidence from rural Kenya. [article]. World Development.

[CR10] Chamberlin J, Jayne TS, Headey D (2014). Scarcity amidst abundance? Reassessing the potential for cropland expansion in Africa. Food Policy.

[CR11] Chamberlin, J., Jayne, T. S., & Snapp, S. (2020). The role of active soil carbon in influencing the profitability of fertilizer use: Empirical evidence from smallholder maize plots in Tanzania. *Authorea, Preprints*. 10.22541/au.158678520.09414228. 10.1002/ldr.3940 PMC8251585 34239284

[CR12] Chengula AA, Mdegela RH, Kasanga CJ (2013). Socio-economic impact of Rift Valley fever to pastoralists and agro pastoralists in Arusha, Manyara and Morogoro regions in Tanzania. SpringerPlus.

[CR13] Christiaensen, L., Demery, L., & Paternostro, S. (2003). *Reforms, remoteness and risk in Africa: Understanding inequality and poverty during the 1990s (No. 2003/70)*. UNU-WIDER.

[CR14] De Groote, H., Doss, C., Lyimo, S., & Mwangi, W. (2002). Adoption of maize technologies in East Africa–what happened to Africa's emerging maize revolution. In *FASID Forum V, "Green Revolution in Asia and its Transferability to Africa", Tokyo, December 8–10, 2002.*

[CR15] Duflo E, Kremer M, Robinson J (2008). How high are rates of return to fertilizer? Evidence from field experiments in Kenya. American Economic Review.

[CR16] Duflo E, Kremer M, Robinson J (2011). Nudging farmers to use fertilizer: Theory and experimental evidence from Kenya. American Economic Review.

[CR17] Farrow A, Risinamhodzi K, Zingore S, Delve RJ (2011). Spatially targeting the distribution of agricultural input stockists in Malawi. Agricultural Systems.

[CR18] Haggblade S, Smale M, Kergna A, Theriault V, Assima A (2017). Causes and consequences of increasing herbicide use in Mali. The European Journal of Development Research.

[CR19] Headey D, Stifel D, You L, Guo Z (2018). Remoteness, urbanization, and child nutrition in sub-Saharan Africa. Agricultural Economics.

[CR20] Koussoubé E, Nauges C (2016). Returns to fertiliser use: Does it pay enough? Some new evidence from sub-Saharan Africa. European Review of Agricultural Economics.

[CR21] Langyintuo AS, Mwangi W, Diallo AO, MacRobert J, Dixon J, Bänziger M (2010). Challenges of the maize seed industry in eastern and southern Africa: A compelling case for private–public intervention to promote growth. Food Policy.

[CR22] Lewis, L., & Masinjila, S. (2018). *The future of smallholder farmer support in Tanzania: Where to after the National Agricultural Input Voucher System (NAIVS)*. The African Centre for Biodiversity.

[CR23] Liverpool-Tasie LSO, Omonona BT, Sanou A, Ogunleye WO (2017). Is increasing inorganic fertilizer use for maize production in SSA a profitable proposition? Evidence from Nigeria. Food Policy.

[CR24] Lyimo S, Mduruma Z, de Groote H (2014). The use of improved maize varieties in Tanzania. African Journal of Agricultural Research.

[CR25] Mabaya, E., Mzee, F., Temu, A., & Mugoya, M. (2017). *Tanzania brief 2017 - the African seed access index*. TASAI.

[CR26] Makinde, K., & Muhhuku, F. (2017). Getting improved seeds to smallholder farmers through agro-dealer networks. In *Seeding an African green revolution: The PASS journey* (pp. 89–107). AGRA.

[CR27] Marenya PP, Barrett CB (2009). State-conditional fertilizer yield response on Western Kenyan farms. American Journal of Agricultural Economics.

[CR28] Mather, D., & Ndeytabula, D. (2016). *Assessing the drivers of Tanzania’s fertilizer subsidy programs from 2003–2016: An application of the kaleidoscope model of policy change. Feed the future innovation lab for food security policy*. Michigan State University.

[CR29] Michler JD, Tjernström E, Verkaart S, Mausch K (2019). Money matters: The role of yields and profits in agricultural technology adoption. American Journal of Agricultural Economics.

[CR30] Minten B, Koru B, Stifel D (2013). The last mile(s) in modern input distribution: Pricing, profitability, and adoption. [article]. Agricultural Economics (United Kingdom).

[CR31] Nagarajan, L. (2015) *Impact assessment of the effectiveness of agro-dealer development activities conducted by USAID-AIMS project in Mozambique*. IFDC.

[CR32] National Bureau of Statistics (2010). Preliminary Report from the National Sample Census of Agriculture 2007/08. . Dar es Salaam, Tanzania: The Tanzanian National Bureau of Statistics and the Office of the Chief Government Statistician, Zanzibar, in collaboration with the Ministry of Agriculture, Food Security and Cooperatives, Ministry of Livestock Development and Fisheries, Ministry of Water and Irrigation, Ministry of Agriculture, Livestock and Environment, Zanzibar, Prime Minister's Office, Regional Administration and Local Governments, Ministry of Industries, Trade and Marketing.

[CR33] Ncube, P., Roberts, S. C., & Vilakazi, T. (2016). Regulation and rivalry in transport and supply in the fertilizer industry in Malawi, Tanzania and Zambia. In S. Robert (Ed.), *Competition in Africa: Insights from key industries* (pp. 102–131). HSRC Press.

[CR34] Odame H, Muange E (2011). Can agro-dealers deliver the green revolution in Kenya? [article]. IDS Bulletin.

[CR35] OSM (2021). OpenStreetMap data https://planet.openstreetmap.org.

[CR36] Rutsaert P, Donovan J (2020). Sticking with the old seed: Input value chains and the challenges to deliver genetic gains to smallholder maize farmers. Outlook on Agriculture.

[CR37] Stifel D, Minten B (2008). Isolation and agricultural productivity. Agricultural Economics.

[CR38] Stifel D, Minten B, Koru B (2016). Economic benefits of rural feeder roads: Evidence from Ethiopia. The Journal of Development Studies.

[CR39] Suri T (2011). Selection and comparative advantage in technology adoption. Econometrica.

[CR40] Tamru S, Minten B, Alemu D, Bachewe F (2017). The rapid expansion of herbicide use in smallholder agriculture in Ethiopia: Patterns, drivers, and implications. The European Journal of Development Research.

[CR41] Weiss DJ, Nelson A, Gibson HS, Temperley W, Peedell S, Lieber A, Hancher M, Poyart E, Belchior S, Fullman N, Mappin B, Dalrymple U, Rozier J, Lucas TCD, Howes RE, Tusting LS, Kang SY, Cameron E, Bisanzio D, Battle KE, Bhatt S, Gething PW (2018). A global map of travel time to cities to assess inequalities in accessibility in 2015. Nature.

[CR42] World Bank. (2014). *Tanzania public expenditure review: National agricultural input voucher scheme*. World Bank.

[CR43] Zavale H, Matchaya G, Vilissa D, Nhemachena C, Nhlengethwa S, Wilson D (2020). Dynamics of the fertilizer value chain in Mozambique. Sustainability.

